# Collateral effects of COVID-19 countermeasures on hepatitis E incidence pattern: a case study of china based on time series models

**DOI:** 10.1186/s12879-024-09243-x

**Published:** 2024-03-27

**Authors:** Yajun Qin, Haiyang Peng, Jinhao Li, Jianping Gong

**Affiliations:** Department of Hepatobiliary Surgery, The Second Hospital Affiliated to Chongqing Medical University, Chongqing, P.R. China

**Keywords:** Hepatitis E, Incidence, COVID-19, Forecasting, Computer neural networks

## Abstract

**Background:**

There are abundant studies on COVID-19 but few on its impact on hepatitis E. We aimed to assess the effect of the COVID-19 countermeasures on the pattern of hepatitis E incidence and explore the application of time series models in analyzing this pattern.

**Methods:**

Our pivotal idea was to fit a pre-COVID-19 model with data from before the COVID-19 outbreak and use the deviation between forecast values and actual values to reflect the effect of COVID-19 countermeasures. We analyzed the pattern of hepatitis E incidence in China from 2013 to 2018. We evaluated the fitting and forecasting capability of 3 methods before the COVID-19 outbreak. Furthermore, we employed these methods to construct pre-COVID-19 incidence models and compare post-COVID-19 forecasts with reality.

**Results:**

Before the COVID-19 outbreak, the Chinese hepatitis E incidence pattern was overall stationary and seasonal, with a peak in March, a trough in October, and higher levels in winter and spring than in summer and autumn, annually. Nevertheless, post-COVID-19 forecasts from pre-COVID-19 models were extremely different from reality in sectional periods but congruous in others.

**Conclusions:**

Since the COVID-19 pandemic, the Chinese hepatitis E incidence pattern has altered substantially, and the incidence has greatly decreased. The effect of the COVID-19 countermeasures on the pattern of hepatitis E incidence was temporary. The incidence of hepatitis E was anticipated to gradually revert to its pre-COVID-19 pattern.

**Supplementary Information:**

The online version contains supplementary material available at 10.1186/s12879-024-09243-x.

## Background

Hepatitis E is a major public health problem and one of the most frequent causes of acute hepatitis worldwide. The global burden of hepatitis E is high; every year, there are approximately 20 million hepatitis E virus (HEV) infections, leading to approximately 3.3 million symptomatic cases of hepatitis E and an estimated 70 000 deaths [1, 2]. HEV is primarily transmitted through the fecal–oral route, and can also be spread through blood and vertical transmission. Although the infection is usually self-limiting, it may cause chronic hepatitis and rapidly progress to cirrhosis in patients with immunodeficiency [3]. In pregnancy, HEV infection can progress into fulminant hepatitis (acute liver failure), which carries a high risk of death; the threat to maternal health and subsequently the fetus surpasses that of hepatitis B, with a mortality rate as high as 25% for third-trimester HEV-infected pregnant women [4, 5]. Accordingly, it is crucial to monitor and analyze the incidence pattern of hepatitis E.

Since the COVID-19 pandemic, there has been a series of non-pharmaceutical interventions, including social distance control, limiting personal movement restriction, enhancing personal protection. These measures not only prevent the spread of COVID but also guard against other infectious diseases. Several studies indicated that COVID-19 prevention measures can partially reduce tuberculosis, scarlet fever, rubella, diphtheria, pertussis, etc. [6–9]. Therefore, it is necessary to study the impact of the COVID-19 countermeasures on hepatitis E to guide prevention and control strategies for these diseases.

In this context, we analyzed the incidence of hepatitis E in China to determine the differences in the pattern of hepatitis E before and after the COVID-19 outbreak and accessed the application of time series models in analyzing the pattern of hepatitis E incidence. Here, we sought to reveal the effect of the COVID-19 pandemic on the pattern of hepatitis E incidence.

## Materials and Methods

### Data Source

Information on the incidence of hepatitis E in China (Table S1) was obtained from *the Overview of the National Legal Infectious Disease Epidemic Situation* published by the National Bureau of Disease Control and Prevention (China, http://www.nhc.gov.cn/) online and collected every month from January 2013 to February 2023. According to Chinese laws, all medical institutions are required to promptly report statutory infectious diseases including hepatitis E through the online reporting system. If the conditions for online reporting are not available, they should report to the relevant local government authorities. The reported data is subject to legal review and quality control by relevant government departments in China, supported by a comprehensive system of supervision and error correction. The data source is authoritative, and the contents are reliable.

### Methods

Our pivotal idea was that a pre-COVID-19 incidence model (fitted with monthly incidence data from 2013 to 2019) and its forecast values should be in accordance with the incidence pattern before the COVID-19 outbreak. Since the COVID-19 outbreak was the only new variable introduced, the deviation between forecast values and actual values should theoretically reflect the effect of COVID-19 countermeasures. We chose 2 models that have been widely applied in incidence forecasting over the years, namely the seasonal autoregressive integrated moving average (SARIMA) model and the Holt-Winters model. Additionally, we have embraced the emerging technology in the fields of mathematics and computer science, the artificial neural network modeling.

The raw data were organized through Excel, while the data processing, model fitting and forecasting, and statistical analysis in this study were performed with *R 4.2.2* (RRID:SCR_001905) with the *forecast*, *tseries*, and *openxlsx* packages. The main functions we used are shown in Table S2. The root mean square error (RMSE) and mean absolute percentage error (MAPE) were adopted to evaluate the errors in model fitting and forecasting. The confidence level in this study was set to 95%.

#### SARIMA model

The seasonal autoregressive integrated moving average (SARIMA) model is a variant of the ARIMA model that introduces seasonal parameters, thus leading to the superior capability to fit time series for diseases with strong seasonality, such as influenza [10]. The model is denoted by$ARIMA\left(p,d,q\right){\left(P,D,Q\right)}_{m}$. In addition to the parameters in the ARIMA model, $P$ denotes the order of the seasonal autoregressive part, $D$ denotes the degree of seasonal first differencing involved, $Q$ denotes the order of the seasonal moving average part, and $m$ denotes the seasonal period (e.g., number of observations per year) [11]. In R, we used the *auto.arima()* function with seasonal parameter set to TRUE to fit the model with the exact maximum likelihood estimation and AIC used to select the optimal model [12] and the *forecast()* function for forecasting.

#### Holt-Winters model

The Holt-Winters exponential smoothing method, proposed by Charles C. Holt & Peter R. Winters in 1960 [13, 14], is a special and modified type of exponential smoothing method that comprehensively measures the smoothness, trend, and seasonality of time series. The elemental thought of the Holt-Winters model is to introduce a seasonal parameter to the quadratic exponential smoothing method, which permits us to decompose time series by the dimension of seasonality. Therefore, to fit the time series, which consists of random variations, linear trends, and seasonality, we could employ the Holt-Winters model. The model is classified into additive seasonal and multiplicative seasonal types as follows.

$$\mathrm{Additive}\;\mathrm{seasonal}\;\mathrm{model}:{\widehat Y}_{t+h}=a_t+hb_t+s_{t-p+1+\left(h-1\right)modp};a_t=\alpha(Y_t-s_{t-p})+\left(1-\alpha\right)\left(a_{t-1}+b_{t-1}\right);b_t=\beta(a_t-a_{t-1})+(1-\beta)b_{t-1};s_t=\gamma(Y_t-a_t)+(1-\gamma)s_{t-p}.$$

$$\mathrm{Multiplicative}\;\mathrm{seasonal}\;\mathrm{model}:{\widehat Y}_{t+h}=\left(a_t+hb_t\right)s_{t-p+1+\left(h-1\right)modp};a_t=\alpha(Y_t/s_{t-p})+(1-\alpha)(a_{t-1}+b_{t-1});b_t=\beta(a_t-a_{t-1})+(1-\beta)b_{t-1};s_t=\gamma(Y_t/a_t)+\left(1-\gamma\right)s_{t-p}.$$

The time series is decomposed into three components: ${a}_{t}$, the level statistic,${b}_{t}$, the trend statistic, and${s}_{t}$, the seasonal statistic; ${s}_{t}$ is added to the trend and level components in the additive model and multiplied by the trend and level components in the multiplicative model. $p$ denotes the seasonal period.$\alpha$,$\beta$, and $\gamma$ are the level smoothing factor, trend smoothing factor, and seasonal smoothing factor, which balance the weights of current values and preceding values in the level, trend, and seasonal parts, respectively. When the smoothing factor approaches 0, the weights of the current values shrink, the time series becomes more stationary, and the effective forecast period becomes longer in sync. In R, we used the *HoltWinters()* function to fit the model, selected optimal parameters by minimizing the squared error [11], and used the *forecast()* function for forecasting.

#### NNAR model

Artificial neural networks are hierarchical network structures designed by mimicking the simple mathematical structure of the brain, consisting of the input layer (bottom layer), the output layer (top layer), and the hidden layer (middle layer). The network can establish complex nonlinear relationships between independent and dependent variables. For time series, the lagged values can be used as the inputs of the neural network, which is called neural network autoregression (NNAR). The model is denoted by$NNAR{(p,P,k)}_{m}$, where $p$ denotes the optimal lag order of the linear AR process, $P$ denotes the optimal lag order of the seasonal AR process, and $k$ denotes the number of nodes in the hidden layer. In R, we used the *nnetar()* function to fit the model. For seasonal time series, the default values are $P$ = 1, and $p$ is chosen from the optimal linear model fitted to the seasonally adjusted data. If $k$ is not specified, it is set to $k=(p+P+1)/2$ (rounded to the nearest integer) [11]. We used the *forecast()* function for forecasting.

## Results

### Analysis of the pattern of hepatitis E incidence before the COVID-19 outbreak

**Fig. 1:**
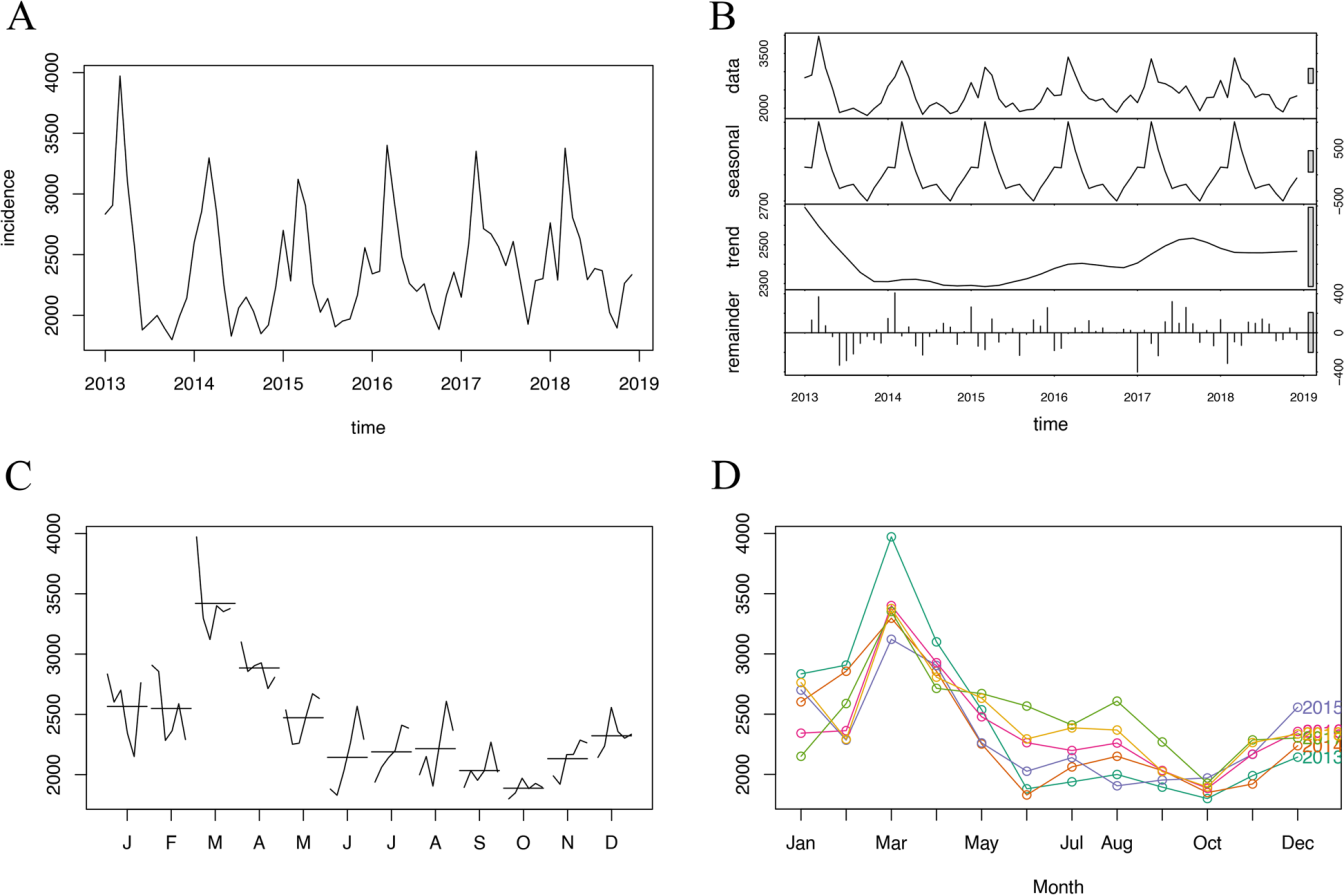
The monthly incidence of hepatitis E in China from January 2013 to December 2018 **A** raw data; **B** original series, seasonal components, trend components, and irregular components; **C** month plot; **D** season plot

**Fig. 2:**
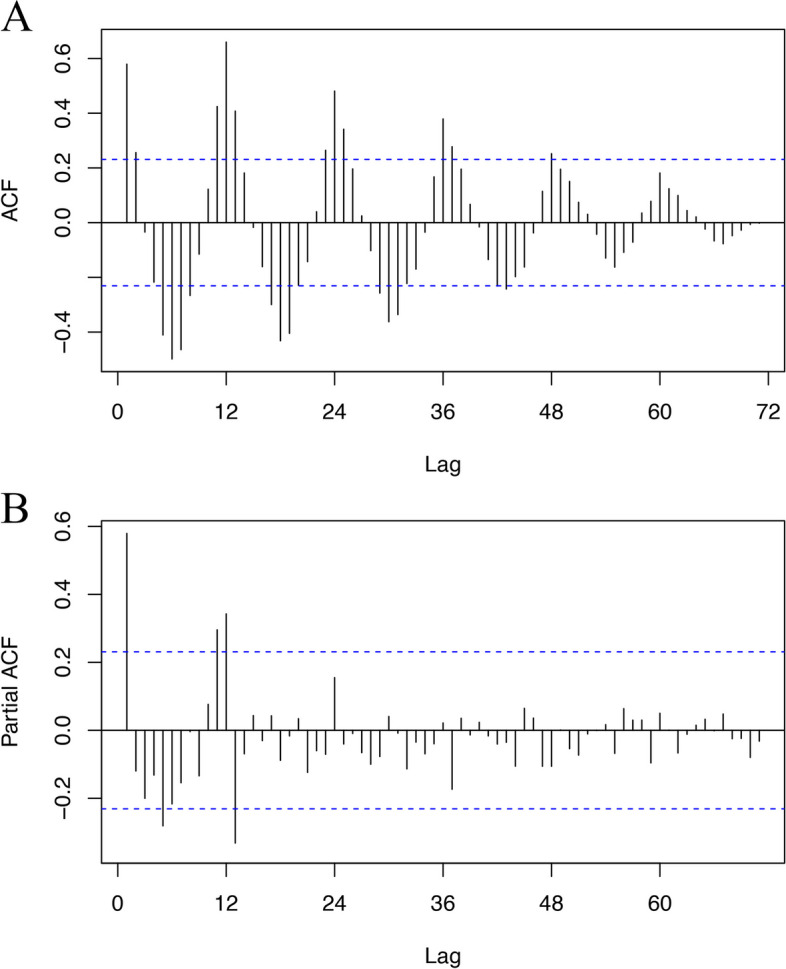
The autocorrelation plot and partial autocorrelation plot with original incidence series from 2013 to 2018 (**A**) autocorrelation; (**B**) partial autocorrelation

Typically, the monthly incidence of hepatitis E in China from 2013 to 2018 was assumed to be trend stationary and nonrandom (Fig. 1), which was verified by the Box-Ljung test (*χ*^*2*^ = 252.73, *df* = 24, *p* < 0.001) and the augmented Dickey-Fuller test (*Dickey-Fuller* = -5.9816, *lag order* = 4, *p* < 0.01). The difference analysis of the original series showed that *ndiff* = 0; hence, there was no demand for differencing before modeling. In the estimate of the autocorrelation function (ACF) and partial autocorrelation function (PACF) with the original series (Fig. 2), the autocorrelation and partial autocorrelation function reached their maximum values (which exceeded the boundary) when *lag* = 12. Moreover, the ACF exhibited the classic pattern of the sinusoid (with a period of approximately 12) gradually converging on 0, indicating that the series had strong seasonality with a period of 12 (months), which was consistent with our visual observation. To further confirm our observations, we decomposed the incidence series into seasonal, trend, and irregular components using LOESS smoothing (Fig. 1). The results showed significant seasonality and the absence of a trend. Furthermore, the month plot (for each month, a subseries was plotted) and season plot (data were plotted against the months in separate years) are shown in Fig. 1. The results illustrated that there was no noteworthy trend among every Jan. Feb. … Dec., but there was convincing seasonality with a peak in March and a trough in October annually. Analysis of the seasonal components by LOESS smoothing showed the same conclusion (highest value 1008.19 in March; lowest value -499.01 in October), and there were higher levels from January to May (seasonal components > 0) than from June to December (seasonal components < 0). Since the pattern was steadily repetitive each year, we suggested that there was a specific and constant pattern of hepatitis E incidence in China before the COVID-19 outbreak.

In summary, before the COVID-19 outbreak, the pattern of hepatitis E incidence in China was overall stationary and seasonal, with a peak in March, a trough in October, and higher levels in winter and spring than in summer and autumn, annually.

### Model assessment

To assess those previously selected mathematical methods’ ability to describe the pattern of hepatitis E incidence before the COVID-19 outbreak, we fitted and modeled the monthly incidence from 2013 to 2018 in China with these methods. As a result, we obtained 3 models: $SARIMA\left(\mathrm{0,0},0\right){\left(\mathrm{0,1},0\right)}_{12}$; additive seasonal Holt-Winters model with trend; and $NNAR{(\mathrm{1,1},2)}_{12}$. We subsequently forecasted the monthly incidence in 2019. Determined by the accuracy of forecasting, the NNAR model outperformed the other models. The detailed process is as follows.

#### Model fitting and effect analysis

We calculated the fitting RMSEs and the fitting MAPEs of the 3 models (Table 1). The SARIMA, Holt-Winters and NNAR models had RMSEs of approximately 180–210 and MAPEs of approximately 6%-7%, which indicates a high degree of accuracy, according to the standards set by Lewis C.D.'s previous research [15]. We conducted the Box-Ljung test and augmented the Dickey-Fuller test for each model’s fitting series (Table 2), and all obtained *p* < 0.05, indicating that the models’ fitting series were nonrandom and stationary. In addition, we conducted the Box-Ljung test for each model’s fitting residuals (Table 2). All models obtained *p* > 0.05, indicating that the residuals were independent and the fitting was accurate.

**Table 1:** The fitting RMSEs, the fitting MAPEs, the forecast RMSEs, and the forecast MAPEs from models (with raw data from 2013 to 2018)

	**Model**	**SARIMA**	**Holt-Winters**	**NNAR**
**Fitting with raw data from 2013 to 2018**	RMSE	206.23	198.63	189.58
	MAPE	6.03%	6.62%	6.26%
**Forecasts from Jan. to Dec. 2019**	RMSE	169.24	165.99	172.36
	MAPE	5.46%	5.44%	5.32%
**Forecasts from Jan. to Nov. 2019**	RMSE	114.37	115.59	123.28
	MAPE	3.80%	3.87%	3.71%

*MAPE* mean absolute percentage error, *RMSE* root mean square error

**Table 2:** Results of the Box-Ljung test for fitting series, the augmented Dickey-Fuller test for fitting series, the Box-Ljung test for fitting residuals, and the Kolmogorov–Smirnov test for fitting residuals (with raw data from 2013 to 2018)

			**SARIMA**	**Holt-Winters**	**NNAR**
**Fitting series**	Box-Ljung test (df = 24)	*χ*^*2*^	309.47	278.12	243.01
		*p*	< 0.001	< 0.001	< 0.001
	augmented Dickey–Fuller test (lag order = 4)	*D-F*	-5.78	-4.92	-5.17
		*p*	< 0.01	< 0.01	< 0.01
**Fitting residuals**	Box-Ljung test (df = 24)	*χ*^*2*^	34.05	30.57	23.54
		*p*	0.0838	0.1665	0.488
	Kolmogorov–Smirnov test	*D*	0.1352	0.0942	0.0676
		*p*	0.1437	0.6269	0.9296

*χ*^*2*^, *D-F* and *D*: the test statistic; *p* *p*-value

We also estimated the ACF and PACF with the fitting residuals (Fig. S1, Fig. S2). In all 3 models, the ACF/PACF exceeded the boundary at a few lags. Subsequently, we plotted the fitting residuals, their histograms (Figure S2), and their normal quantile‒quantile plots (Q-Q plots, Figure S1). The residuals approximately fit the 0-mean normal distribution, but there were several deviating values. Accordingly, we further conducted the exact one-sample Kolmogorov‒Smirnov test for those residuals (Table 2) and obtained *p* > 0.05 for every model, indicating that those residuals could still fit the 0-mean normal distribution and that the deviations were random.

In summary, we concluded that the SARIMA, Holt-Winters, and NNAR methods performed smoothly in fitting the hepatitis E incidence series from 2013 to 2018.

#### Model forecasting and effect analysis

**Fig. 3:**
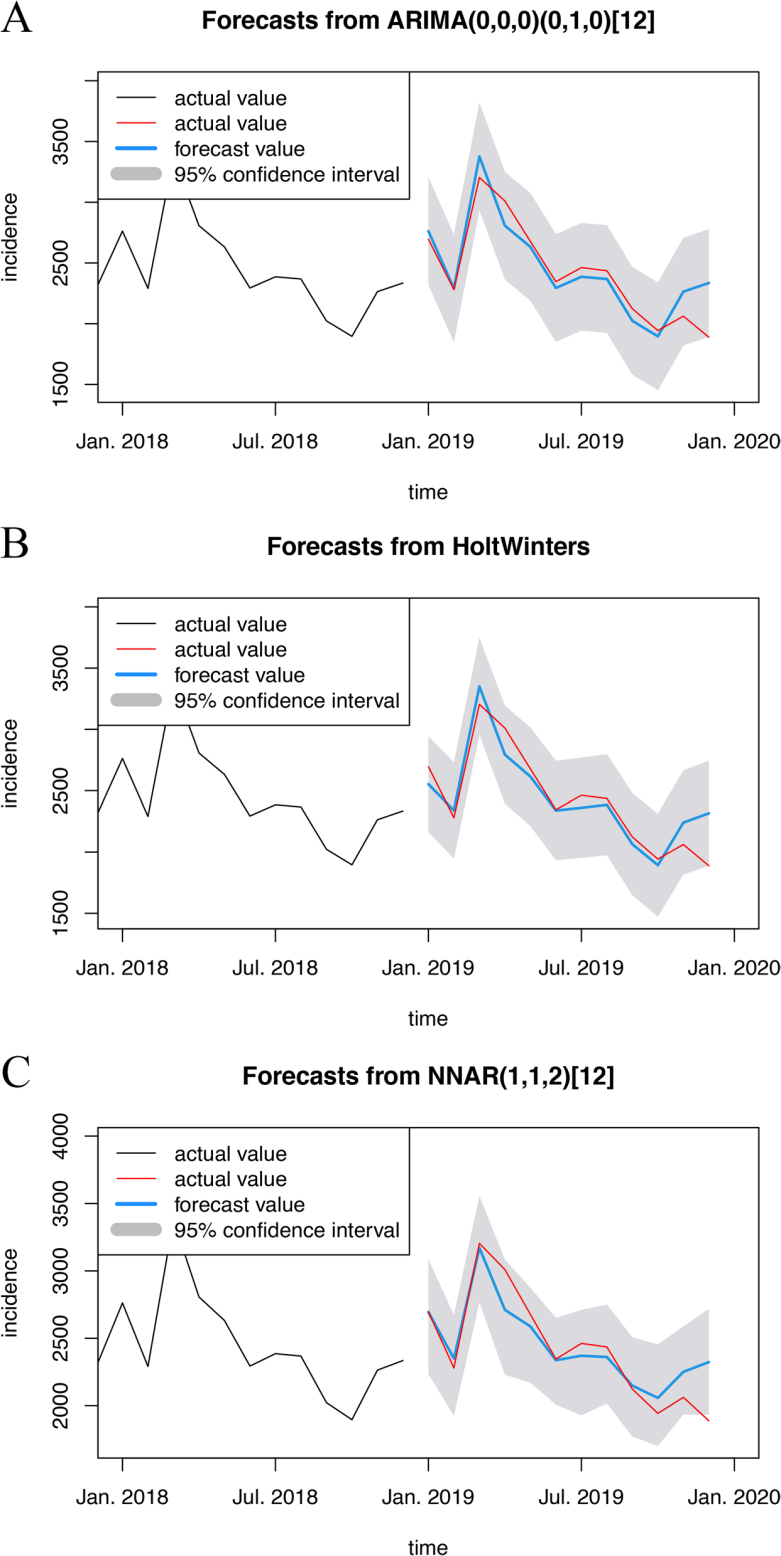
The forecasts (in 2019) from models (with raw data from 2013 to 2018) **A** SARIMA; **B** Holt-Winters; **C** NNAR. The black lines refer to input to the model. The red lines refer to the actual values which were not used for model fitting

To cross-check, we employed the established models to forecast the incidence in 2019 and contrast this with actual values. To evaluate errors in forecasting, we calculated their RMSEs and MAPEs (Table 1). The NNAR model acquired the forecasting MAPE of 5.32%, which was significantly lower than those acquired by the other models. The forecast values of those 3 models from January to November 2019 were all within the 95% confidence interval (CI) of the models. However, the forecast in December was noticeably worse than those of the previous 11 months, exceeding the 95% CI in all 3 models. The exceedances were -0.18%, -0.0039%, and -5.5% of the forecast values (Fig. 3). Therefore, we calculated the RMSEs and MAPEs of those 3 models from January to November 2019 (Table 1). The RMSEs and MAPEs of all 3 models decreased to 120 and 3%, indicating the forecast deviation in December 2019, possibly attributed to the inchoate influence of COVID-19 on the pattern of hepatitis E incidence. Meanwhile, the forecasting MAPE of the NNAR model was still the lowest.

After comprehensive consideration, the SARIMA, Holt-Winters, and NNAR methods were optimal for describing the pattern of hepatitis E incidence before the COVID-19 outbreak. Among them, the NNAR method exhibited the best performance.

### Analysis of the incidence pattern of hepatitis E after the COVID-19 outbreak

We exploited the SARIMA, Holt-Winters, and NNAR methods to fit and model the monthly incidence of hepatitis E in China from 2013 to 2019 and obtained the following models: $SARIMA\left(\mathrm{0,0},0\right){\left(\mathrm{0,1},0\right)}_{12}$, additive seasonal Holt-Winters model with the trend, and $NNAR{(\mathrm{1,1},2)}_{12}$ (same type as in 3.2).

We calculated the fitting RMSEs and fitting MAPEs of the SARIMA model, Holt-Winters model, and NNAR model. The RMSEs were 201.36, 194.01, and 184.46, while the MAPEs were 5.94%, 6.38%, and 6.05%, respectively, indicating the smooth performance of fitting. We analyzed the fitting effect by the same process as in 3.2.1 and received the appropriate results as follows.

We conducted the Box-Ljung test for each model’s fitting series, and all obtained *p* < 0.05, indicating that the models’ fitting series were nonrandom. (SARIMA: *χ*^*2*^ = 348.92, *df* = 24, *p* < 0.001; Holt-Winters: *χ*^*2*^ = 334.32, *df* = 24, *p* < 0.001; NNAR: *χ*^*2*^ = 297.97, *df* = 24, *p* < 0.001).

We conducted the augmented Dickey-Fuller test for each model’s fitting series, and all obtained *p* < 0.05, indicating that the models’ fitting series were stationary. (SARIMA: *Dickey-Fuller* = -6.3587, *lag order* = 4, *p* < 0.01; Holt-Winters: *Dickey-Fuller* = -6.0352, *lag order* = 4, *p* < 0.01; NNAR: *Dickey-Fuller* = -5.7641, *lag order* = 4, *p* < 0.01).

We conducted the Box-Ljung test for each model’s fitting residuals, and all obtained *p* > 0.05, indicating that the residuals were independent and the fitting was accurate. (SARIMA: *χ*^*2*^ = 32.778, df = 24, p = 0.1089; Holt-Winters: *χ*^*2*^ = 29.361, df = 24, *p* = 0.2068; NNAR: *χ*^*2*^ = 27.452, *df* = 24, *p* = 0.2838).

**Fig. 4:**
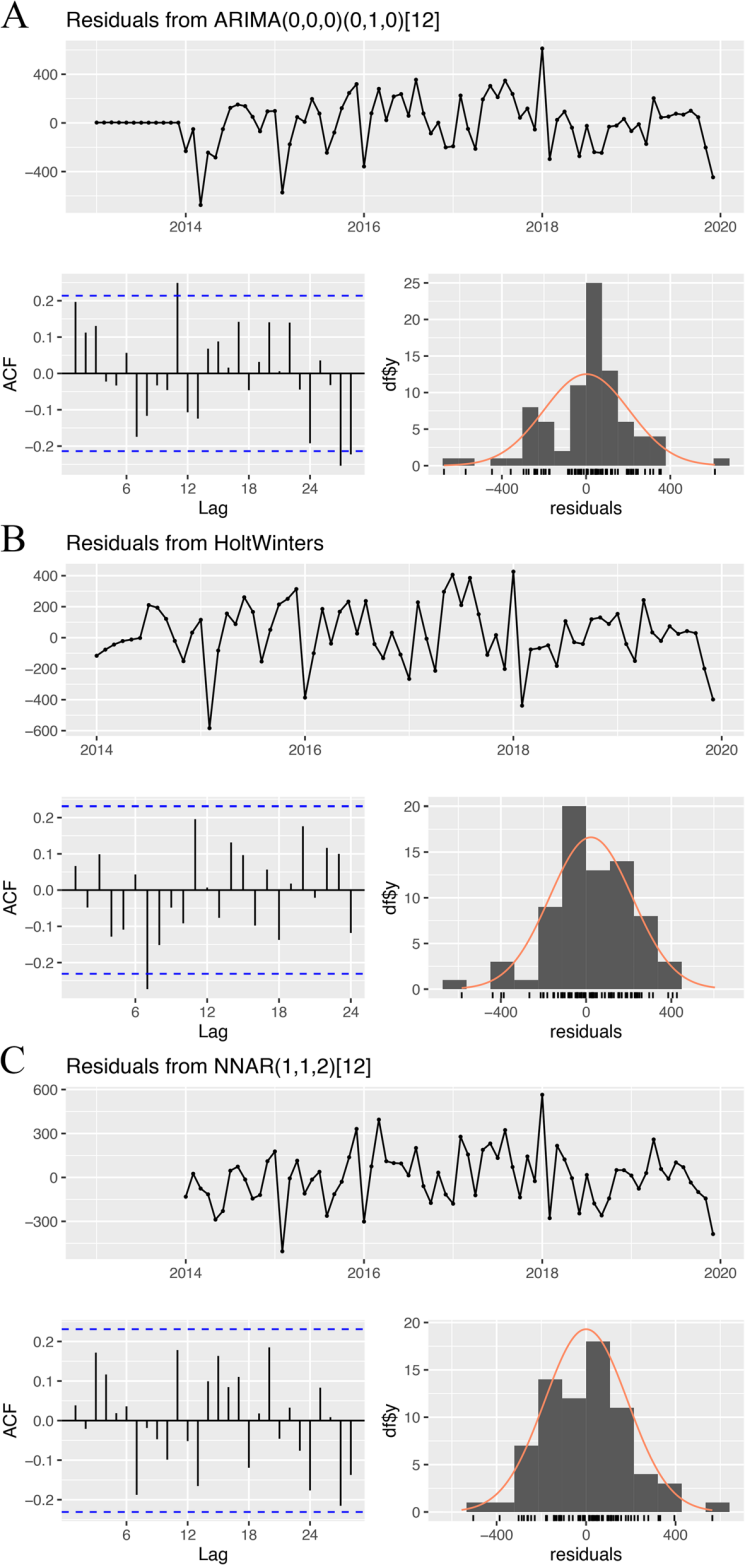
The fitting residuals (with raw data from 2013 to 2019), their autocorrelation plots, and their histograms **A** SARIMA; **B** Holt-Winters; **C** NNAR

We estimated the ACF and PACF with the fitting residuals (Fig. S3, Fig. 4). In all 3 models, the ACF and PACF exceeded the boundary at a few lags.

We plotted the fitting residuals, their histograms (Fig. 4), and their normal quantile‒quantile plots (Q-Q plots, Fig. S3). The residuals approximately fit the 0-mean normal distribution, but there were several deviating values.

We further conducted the exact one-sample Kolmogorov‒Smirnov test for those residuals and obtained *p* > 0.05 for all 3 models, indicating that those residuals could still fit the 0-mean normal distribution and that the deviations were random. (SARIMA: *D* = 0.13065, *p* = 0.1136; Holt-Winters: *D* = 0.062714, *p* = 0.9226; NNAR: *D* = 0.053952, *p* = 0.9775).

**Fig. 5:**
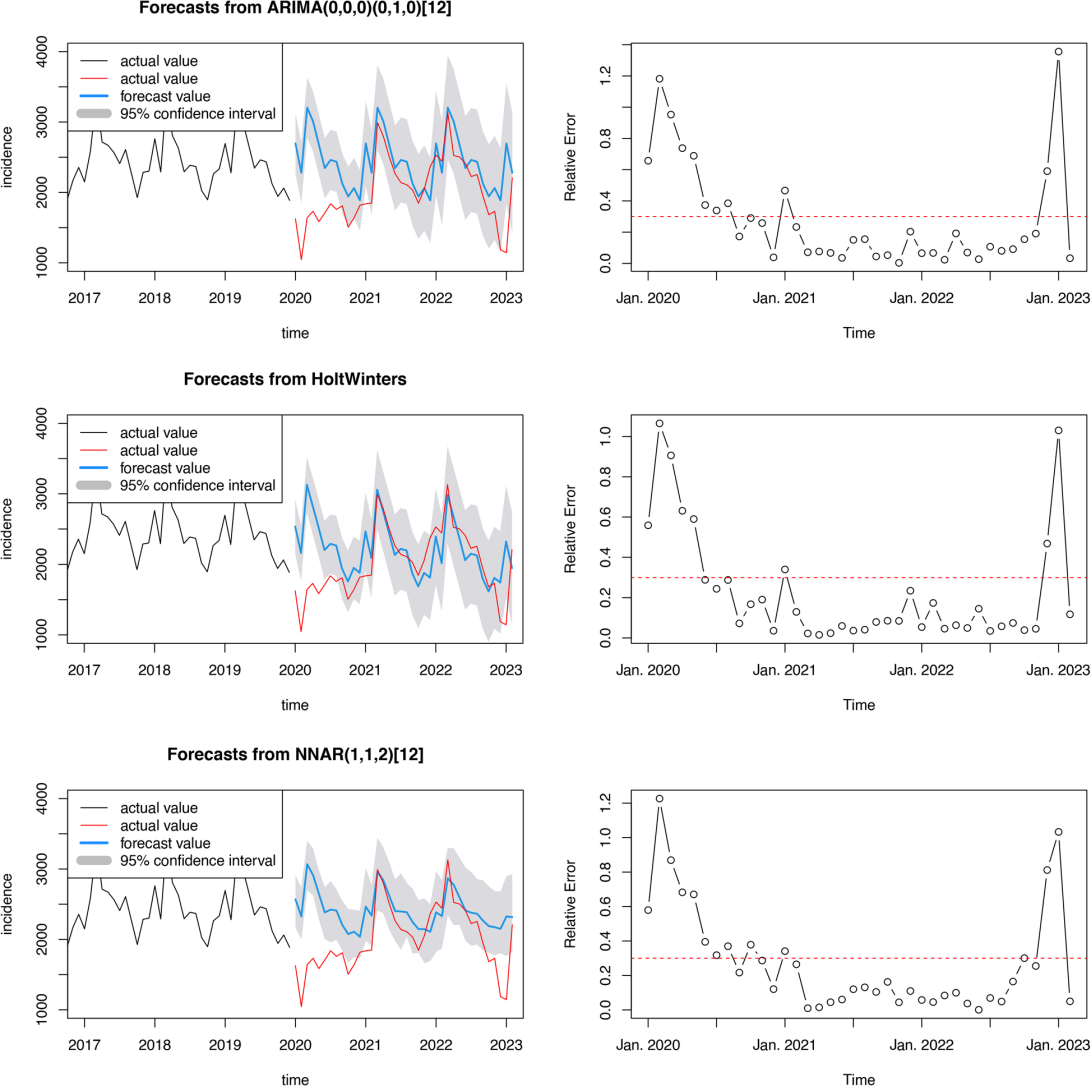
The forecasts (in from January 2020 to February 2023) and relative errors **A** SARIMA; **B** Holt-Winters; **C** NNAR. The black lines refer to input to the model. The red lines refer to the actual values which were not used for model fitting

To cross-check, we employed the established models to forecast the incidence from January 2020 to February 2023 (Fig. 5). A mass of forecast values outdistanced the 95% CI of the models. There were enormous deviations between the incidence pattern before and after the COVID-19 outbreak, which concretely manifested as a far diminished incidence.

To further investigate the deviation of the incidence pattern before and after the COVID-19 outbreak and explore the possible causes, we calculated the monthly relative error of the forecast (Fig. 5). There were pronounced error peaks in February 2020 and January 2023. Based on the level of relative error, we divided the time series into 5 stages: January 2020 to May 2020, June 2020 to February 2021, March 2021 to November 2022, December 2022 to January 2023, and February 2023. We calculated the RMSE and MAPE for each stage (Table 3). Notably, the forecast errors reached extremely large values in the first stage, gradually decreased in the second and third stages, reached a maximum in the fourth stage and were finally reduced to quite small values in the last stage. Moreover, the forecast values in the third and fifth stages were all within the 95% CI of the models, while the forecast errors approached the level before the COVID-19 outbreak (as in 3.2.1), indicating effective forecasting. In summary, after the COVID-19 pandemic, the pattern of hepatitis E incidence in China changed from January 2020 to May 2020 and from December 2022 to January 2023, while in other periods, the incidence pattern was in accordance with the pre-COVID-19 pattern, which meant that the effect of COVID-19 on the incidence pattern might be temporary.

**Table 3:** The forecast RMSEs and the MAPEs from models (with raw data from 2013 to 2019) in 5 stages

	**Stage**	**SARIMA**	**Holt-Winters**	**NNAR**
**Forecasting MAPE**	Jan. 2020 to May.2020	84.37%	75.03%	80.55%
	Jun. 2020 to Feb. 2021	28.40%	19.53%	29.90%
	Mar. 2021 to Nov. 2022	9.17%	6.98%	9.36%
	Dec. 2022 to Jan. 2023	97.32%	74.94%	92.24%
	In Feb. 2023	3.31%	11.75%	4.98%
**Forecasting RMSE**	Jan. 2020 to May.2020	1259.90	1126.58	1190.66
	Jun. 2020 to Feb. 2021	542.20	385.02	537.57
	Mar. 2021 to Nov. 2022	240.09	203.50	234.34
	Dec. 2022 to Jan. 2023	1203.54	921.74	1078.16
	In Feb. 2023	73.00	259.32	109.98

*MAPE* mean absolute percentage error, *RMSE* root mean square error

## Discussion

Constructing time series models to analyze data and forecast is currently widely performed in various fields, such as economics, energy science, social issues, industrial quality control, geophysics, environmental science, and aeronautics [16–23]. In the field of public health, time series forecast methods are mainly applied to forecast disease burden, disease incidence, population level, age structure, etc. [24–26].

Among the existing methods for time series analysis and forecasting, the ARIMA/SARIMA models are the most widely used and are mainly used for analyzing stationary series with a linear trend. Before modeling, we can convert an explosive series into a stationary series by differencing. The ARIMA/SARIMA model relies on a large scale of uninterrupted data; therefore, the model accuracy may not be ideal in the case of a few deficiencies or outliers [27]. With the seasonal parameter introduced, the Holt-Winters exponential smoothing method would be able to describe the seasonality of time series. When fitting seasonal series with a linear trend, the Holt-Winters model is preferred. The NNAR model possesses hierarchical network structures, constructing complex nonlinear relationships between a response variable and its predictors. One of its superiorities is to fit nonlinear and explosive series. The artificial neural network is an inexplicable black box at this stage, as is the human brain. From another perspective, it could describe not only seasonality, trend, periodism, the holiday effect, etc., but also other ambiguous effects. Therefore, the NNAR model usually outperforms others in forecasting [28].

In previously published studies, some scholars have fitted and forecasted time series by different mathematical methods, whereas most of them only focused on fitting, forecasting, and performance evaluation. In this context, we innovatively applied the forecast model to the analysis of incidence pattern transformation. Our basic thought was to exploit a pre-COVID-19 incidence model to forecast incidence (representing the pre-COVID-19 incidence pattern) and compare it with the actual incidence after the COVID-19 outbreak (representing the post-COVID-19 incidence pattern). Since the COVID-19 outbreak was the only new variable introduced, the deviation between forecast values and actual values should theoretically reflect the effect of COVID-19 countermeasures.

Initially, we analyzed the hepatitis E incidence series before the COVID-19 outbreak. We suggested a specific and constant pattern of hepatitis E incidence in China before the COVID-19 outbreak. The pattern was overall stationary and seasonal, with a peak in March, a trough in October, and higher levels in winter and spring than in summer and autumn, annually, which was consistent with the findings of a previous study by Wei S. et al. [29].

After assessment, we exploited the SARIMA, Holt-Winters, and NNAR methods to construct pre-COVID-19 models of hepatitis E incidence from 2013 to 2019. According to Lewis C.D., a model whose MAPE is less than 10% indicates a high degree of accuracy [15]. In our models, the fitting MAPEs were 6–7% and forecast MAPEs were 3–4%, pronouncedly lower than the models in Li Z.’s work [30].

Subsequently, we exploited the pre-COVID-19 models to forecast the incidence from January 2020 to February 2023. There was enormous forecast deviation, manifested as significantly lower forecast values than the actual values (with MAPEs of approximately 90%).

Among the influential policies on COVID-19 from the Chinese government, there were 2 critical events: in January 2020, the central government of China implemented the emergency epidemic prevention and control policy, while the first-level public health emergency response was activated in more than 30 provinces and cities, and the lockdown began in Wuhan [31]; in December 2022, the National Health Commission announced the management downgrading of COVID-19 from Class A to Class B (in accordance with the national law on infectious disease prevention and treatment) [32]. According to our results, the forecast deviation reached an extremely large scale from January 2020 to May 2020 (MAPE of approximately 80%) and from December 2022 to January 2023 (MAPE of approximately 90%) during the same periods of the Wuhan lockdown (January 2020 to April 2020) and COVID-19 management downgrading (December 2022 to January 2023).

We assumed that the main reason for the forecast deviation was changes in prevention and control policies. In response, the public reduced unnecessary trips and paid more attention to personal hygiene, in which case transmission of the hepatitis E virus was partially cut off, and some patients with hepatitis E did not see a doctor, leading to a significant decrease in incidence. From another perspective, this also indicated a high degree of public credibility of the Chinese government, as the public actively responded to government policies. Moreover, the forecast from March 2021 to November 2022 and February 2023 was quite precise, and all the forecast values were within the 95% CI of those models. These results indicated that during those periods, the effects of COVID-19 countermeasures on the incidence of hepatitis E were small. Therefore, we believe that the effects of the COVID-19 countermeasures on the incidence of hepatitis E may be temporary and based on the public’s response to government policies.

As a result of the absence of suitable quantitative metrics for evaluating COVID-19 policies, this assumption cannot be considered robust evidence. It is imperative to identify quantifiable indicators of the sociological effect of COVID-19, establish corresponding sequences, and construct systematic models to verify this hypothesis. Nevertheless, our primary conclusion, that the impacts of COVID-19 are temporary, is not undermined. As COVID-19 policies are still in place, the pattern of hepatitis E incidence was expected to gradually return to the pre-COVID-19 pattern. Based on that conclusion, we could still forecast the incidence of hepatitis E with selected models henceforward. We could also collect incidence data in real time and compare it with the forecast values. When there is a significant deviation (out of the 95% CI), it is important to be vigilant of possible emerging factors that affect the incidence (epidemic outbreak, etc.). In addition, while collecting incidence data, we can feed new data back into the models to continuously optimize the parameters, enabling the models to update and learn (to adapt to new situations).

## Conclusion

In conclusion, our study constructed efficient time series models to depict the hepatitis E incidence pattern and examined the effect of the COVID-19 countermeasures on this pattern. This effect appeared to be transitory in nature. Since the incidence of hepatitis E was anticipated to gradually revert to its pre-COVID-19 pattern, there is a viable opportunity to establish an epidemiological surveillance and alert system using these time series models. To ascertain the validity of this hypothesis, further studies are essential.

### Supplementary Information

**Supplementary Material 1.**

**Supplementary Material 2.**

**Supplementary Material 3.**

**Supplementary Material 4.**

**Supplementary Material 5.**

## Data Availability

The data that support the findings of this study were published by the National Bureau of Disease Control and Prevention (China, http://www.nhc.gov.cn/) online. The *R* codes are available from the corresponding author on reasonable request.

## Abbreviations

HEV: Hepatitis E virus
SARS-CoV-2: Severe acute respiratory syndrome coronavirus 2
RMSE: Root mean square error
MAPE: Mean absolute percentage error
AIC: Akaike information criterion
SARIMA: Seasonal autoregressive integrated moving average
LLR: Log-likelihood ratio
NNAR: Neural network autoregression
ACF: Autocorrelation function
PACF: Partial autocorrelation function
Q-Q plots: Quantile‒quantile plots
CI: Confidence interval
