# Supplementary material for: Collateral effects of COVID-19 countermeasures on hepatitis E incidence pattern: a case study of china based on time series models
Source: BMC Infect Dis. 2024 Mar 27;24:355. doi: 10.1186/s12879-024-09243-x (PMC10967115; doi:10.1186/s12879-024-09243-x)
Supplement: Supplementary file 2 — Supplementary Material 2. [file 12879_2024_9243_MOESM2_ESM.docx]

**Figure S2. The fitting residuals (with raw data from 2013 to 2018), their autocorrelation plots, and their histograms** (A) SARIMA; (B) Holt-Winters; (C) NNAR.

A.B. C.
