# Supplementary material for: Collateral effects of COVID-19 countermeasures on hepatitis E incidence pattern: a case study of china based on time series models
Source: BMC Infect Dis. 2024 Mar 27;24:355. doi: 10.1186/s12879-024-09243-x (PMC10967115; doi:10.1186/s12879-024-09243-x)
Supplement: Supplementary file 3 — Supplementary Material 3. [file 12879_2024_9243_MOESM3_ESM.docx]

**Figure S3. The autocorrelation plots, the partial autocorrelation plots, and the normal quantile-quantile plots of fitting residuals (with raw data from 2013 to 2019)** (A) SARIMA; (B) Holt-Winters; (C) NNAR.

1. SARIMA

1. Holt-Winters

1. NNAR
