# Supplementary material for: Collateral effects of COVID-19 countermeasures on hepatitis E incidence pattern: a case study of china based on time series models
Source: BMC Infect Dis. 2024 Mar 27;24:355. doi: 10.1186/s12879-024-09243-x (PMC10967115; doi:10.1186/s12879-024-09243-x)
Supplement: Supplementary file 4 — Supplementary Material 4. [file 12879_2024_9243_MOESM4_ESM.docx]

**Table S1. The monthly incidence of hepatitis E in China from January 2013 to February 2023.**

| **Year** | **Jan** | **Feb** | **Mar** | **Apr** | **May** | **Jun** | **Jul** | **Aug** | **Sep** | **Oct** | **Nov** | **Dec** |
| --- | --- | --- | --- | --- | --- | --- | --- | --- | --- | --- | --- | --- |
| **2013** | 2834 | 2907 | 3972 | 3100 | 2536 | 1880 | 1938 | 1999 | 1894 | 1799 | 1989 | 2143 |
| **2014** | 2602 | 2856 | 3297 | 2856 | 2252 | 1829 | 2062 | 2150 | 2032 | 1849 | 1920 | 2238 |
| **2015** | 2700 | 2284 | 3121 | 2904 | 2261 | 2026 | 2139 | 1905 | 1953 | 1970 | 2166 | 2557 |
| **2016** | 2342 | 2363 | 3401 | 2927 | 2478 | 2263 | 2198 | 2260 | 2031 | 1884 | 2168 | 2356 |
| **2017** | 2150 | 2588 | 3352 | 2714 | 2671 | 2567 | 2410 | 2608 | 2269 | 1927 | 2286 | 2302 |
| **2018** | 2762 | 2291 | 3377 | 2807 | 2632 | 2294 | 2386 | 2368 | 2023 | 1896 | 2264 | 2335 |
| **2019** | 2695 | 2280 | 3204 | 3010 | 2677 | 2346 | 2462 | 2436 | 2123 | 1943 | 2062 | 1888 |
| **2020** | 1626 | 1045 | 1641 | 1732 | 1585 | 1708 | 1839 | 1759 | 1811 | 1506 | 1639 | 1818 |
| **2021** | 1838 | 1849 | 2991 | 2797 | 2509 | 2266 | 2140 | 2109 | 2033 | 1846 | 2055 | 2369 |
| **2022** | 2530 | 2443 | 3131 | 2525 | 2503 | 2411 | 2225 | 2255 | 1946 | 1683 | 1732 | 1187 |
| **2023** | 1144 | 2207 |  |  |  |  |  |  |  |  |  |  |
