# Supplementary material for: Collateral effects of COVID-19 countermeasures on hepatitis E incidence pattern: a case study of china based on time series models
Source: BMC Infect Dis. 2024 Mar 27;24:355. doi: 10.1186/s12879-024-09243-x (PMC10967115; doi:10.1186/s12879-024-09243-x)
Supplement: Supplementary file 5 — Supplementary Material 5. [file 12879_2024_9243_MOESM5_ESM.docx]

**Table S2. Main functions used and their objectives.**

| **Function** | **Objective** |
| --- | --- |
| *ts( )* | to create time-series objects |
| *window( )* | to extract set and test set |
| *ndiffs( )* | to estimate the number of differences required to make a time series stationary |
| *stl( )* | to decompose a time series into seasonal, trend, and irregular components using LOESS |
| *Acf( ), Pacf( )* | to estimate the autocorrelations or partial autocorrelations |
| *Box.test( )* | to conduct the Box-Ljung test |
| *adf.test( )* | to conduct the Augmented Dickey-Fuller test |
| *ks.test( )* | to conduct the one-sample Kolmogorov-Smirnov test |
| *arima( ), HoltWinters( ), nnetar( )* | to fit models to a univariate time series |
| *forecast( ), predict( )* | to produce forecasts from fitted models |
| *plot( ), plot.ts( ), par( ), monthplot( ), seasonplot( ), qqnorm( ), qqline( )* | to visualize the results |
